# Supplementary material for: Functional and Transcriptomic Characterization of Postnatal Maturation of ENS and SIP Syncytium in Mice Colon
Source: Biomolecules. 2023 Nov 23;13(12):1688. doi: 10.3390/biom13121688 (PMC10741935; doi:10.3390/biom13121688)
Supplement: Supplementary file 1 [file biomolecules-13-01688-s001.zip › tables and figures.pdf]

Supplementary Material

Table S1. Primers used for qRT-PCR assay.

**Primer**	**Primer sequences (5' ⟶ 3')**
Scn7a-F	TGAAGCTGTTTGGTCGAAG
Scn7a-R	TCGGAACACGGTCATGTA
Kcna1-F	AAGGGCTCCCGTAGTGTT
Kcna1-R	ACGCTGTCCTCTCTCTGG
Gfap-F	AGCGTGCAGAGATGATGG
Gfap-R	AGTTTGGTGGGCTCCTTG
Pdgfrα-F	ATGACAGCAGGCAGGGCTTCAACG
Pdgfrα-R	CGGCACAGGTCACCACGATCGTTT
c-Kit-F	CGCCTGCCGAAATGTATGACG
c-Kit-R	GGTTCTCTGGGTTGGGGTTGC
Nos1-F	ATCTGTCTCGCCAGCCATCAGCCA
Nos1-R	GGAGCTTTGTGCAGTTTGCCGTCG
P2ry1-F	ACCGAGGTGCCTTGGTCGGT
P2ry1-R	CCGGTCTTGGTCAGGGCACA
Slc18a3-F	TCATGTTCGCCTCCACAGTC
Slc18a3-R	GCTCCTCGGGATACTTGTCG
Scn5a-F	GTTCGGCCACCTCACAG
Scn5a-R	AAAGGGTCCAGGTCTTCCA
Snap25-F	TCATCCGCAGGGTAACA
Snap25-R	GCGATTCTGGGTGTCAAT
Syn2-F	ATTCCCTGCGTGTGATAGA
Syn2-R	GGGGTGTTGACTTGTTGAG
Syt1-F	CTTCTCCCTCCGCTACG
Syt1-R	TTCAGTCTCTTGCCGTTCT
Gapdh-F	GCCGATGCCCCCATGTTTGTGA
Gapdh-R	GGGTGGCAGTGATGGCATGGAC
Sox10-F	AACACATCGCTGCCCCTTTA
Sox10-R	GCAGGCAACCAGAAGCATTG
Ret-F	GCATGTCAGACCCGAACTGG
Ret-R	CGCTGAGGGTGAAACCATCC
Phox2b-F	GCGAGAGTCCAGGTGTGGTT
Phox2b-R	CTCTTTGCTCTCGTCGTCCC
Chat-F	AAAATGGCGTCCAACGAGGA
Chat-R	CCCGGTTGGTGGAGTCTTTTA
S100b-F	CGAGAGGGTGACAAGCACAA
S100b-R	TCCTGCTCCTTGATTTCCTCCA

Table S2. All antibodies used for immunoblotting and immunohistochemistry.

Primary/secondary antibody	Catalog	Supplier	Application (Work concentration)
Anti-GFAP	ab7260	ABCAM	WB (1:5000)
Anti-PDGFRα	D1E1E XP	Cell Signaling Technology	Wes (1:100)
Anti-c-Kit	AF1356	R&D systems	Wes (1:100)
Anti-ANO1	ab53212	ABCAM	Wes (1:100)
Anti-NOS1	SC-5302	Santa Cruz Biotechnology	Wes (1:100)
Anti-P2RY1	ER1914-59	HuaBio Technology	WB (1:1000)
Anti-VAChT / SLC18A3	ab235201	ABCAM	WB (1:1000)
Anti-SK3	ab28631	ABCAM	WB (1:5000)
Anti-γ-ACTIN	GTX101794	Genetex	Wes (1:200)
Anti-β-ACTIN	ab8226	ABCAM	WB (1:10000)
HRP-conjugated Anti-rabbit IgG	AF008	R&D systems	Wes (1:1000), WB (1:5000)
HRP-conjugated Anti-mouse IgG	AF007	R&D systems	WB (1:5000)

Table S3. All drugs used in isometric force recording and CMCs assay.

Drug	Catalog	Supplier	Application (Work concentration)
LNNA	No.0665	Tocris	IFR & CMCs (100.0 μM)
MRS2500	No.2159	Tocris	IFR & CMCs (1.0 μM)
Atropine	A-046	Sigma-Aldrich	IFR & CMCs (1.0 μM)

Supplementary Figure Legends

Figure S1. The differentially expressed genes in the whole colon of young mice. (A) The volcano plot of differentially expressed genes between PW1 and PW3 sample. **(B)** The volcano plot of differentially expressed genes between PW1 and PW5 sample. FC, fold change. The red and blue dots represent significantly up-regulated and down-regulated genes (Log2(FC) ≥ 1.0 or ≤ -1.0, $P < 0.05$).

Figure S2. The gene ontology and pathway enrichment of differentially expressed genes. (**A**), (**B**) and (**C**) The top ten biological processes, cellular components, and molecular function of differentially expressed genes in the colon of PW3 mice, respectively. (**D**) The top five enriched KEGG pathway of differentially expressed genes in the colon of PW3 mice. (**E**), (**F**) and (**G**) The top ten biological processes, cellular components, and molecular function of differentially expressed genes in the colon of PW3 mice, respectively. (**H**) The top five enriched KEGG pathway of differentially expressed genes in the colon of PW5 mice.

Figure S3. The quantitative real time PCR results of representative genes in enteric neuron system. Relative quantitative mRNA level of *Scn5a* **(A)**, *Snap25* **(B)**, *Syn2* **(C)**, *Syt1* **(D),** *Sox10* **(E),** *Phox2b* **(F),** *Ret* **(G),** *Chat* **(H) and** *S100b* **(I)** in the whole colon of PW1, PW3, and PW5 mice. The box graphs shown in panels **A-I** represent mean ± SD. Five samples were used for testing each transcript.

Figure S4. The inhibitory effects of the P2Y1 receptor antagonist MRS2500 on CMC of the young murine colon. A-C: Contractile responses of isolated proximal (P), middle (M), and distal colons (D) treated with MRS2500 (1.0 μM) from PW1 (**A**), PW3 (**B**), and PW5 (**C**) mice. **D**: The CMC frequency of each colon segment treated with MRS2500 from PW1, PW3 and PW5 mice. ND represents not determined. **E**: The CMC amplitude of each colon segment treated with MRS2500 from PW1, PW3, and PW5 mice. The bar graphs shown in panel **D** and **E** represent mean ± SE (n = 6). "n" means the number of animals. $^{*}P < 0.05$.

Fig. S1

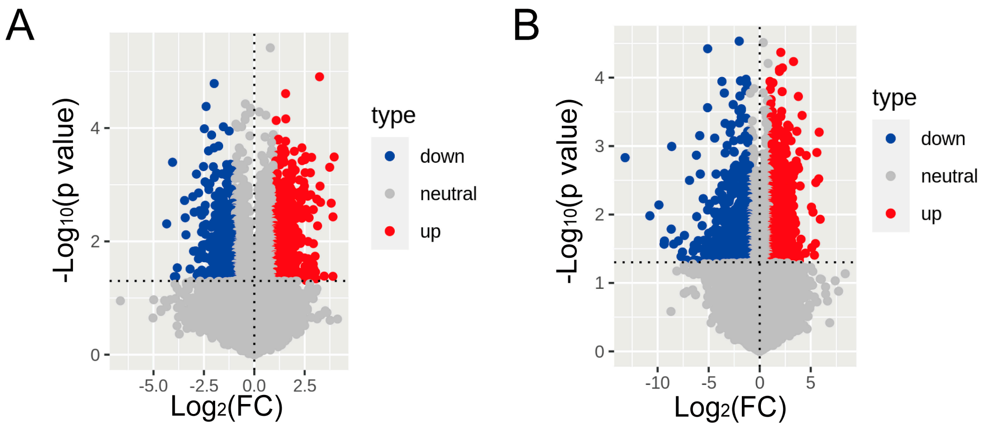
A
-Log10(p value)
Log2(FC)
type
down
neutral
up
B
-Log10(p value)
Log2(FC)
type
down
neutral
up

Fig. S2

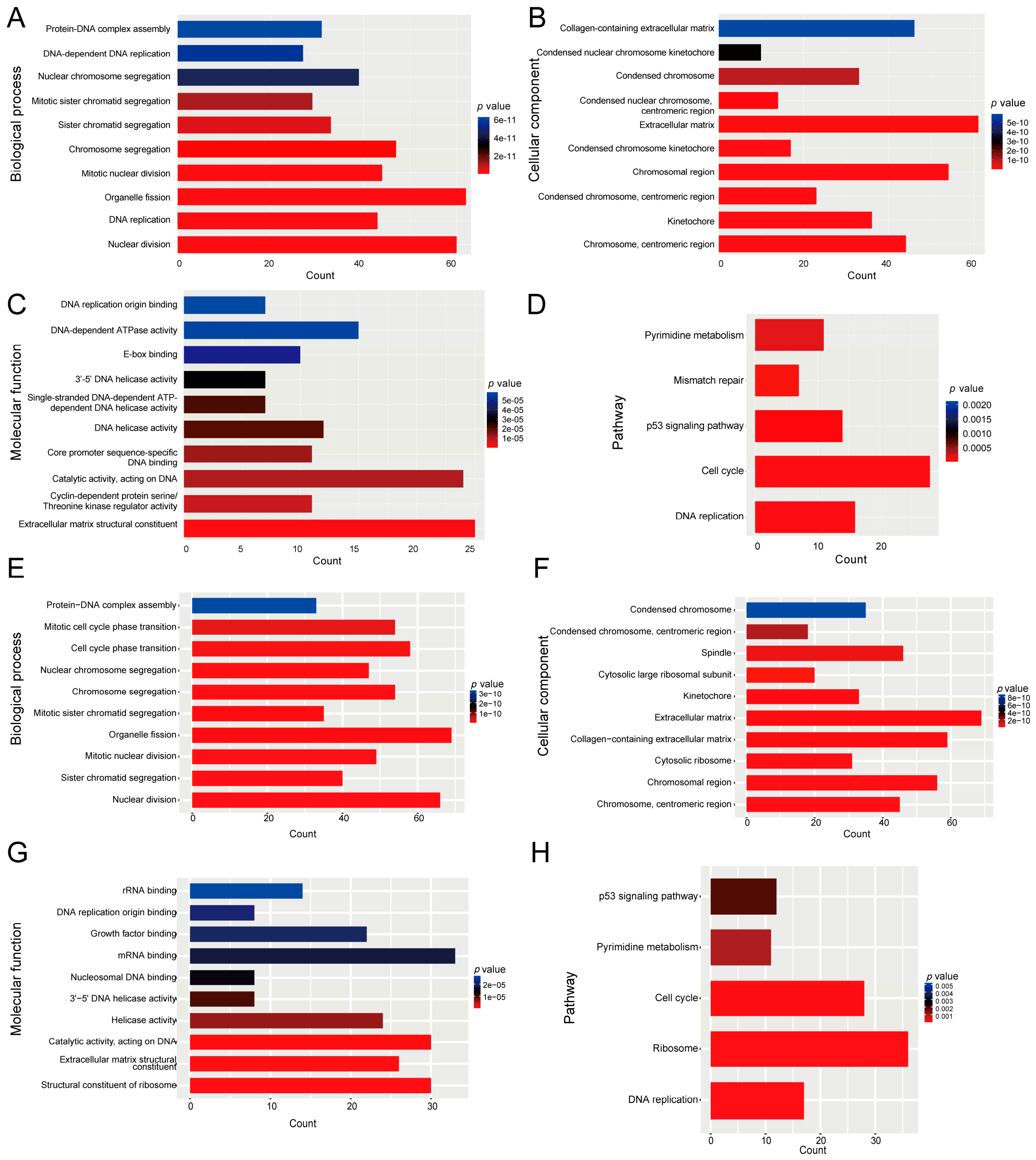
A
Biological process
Protein-DNA complex assembly
DNA-dependent DNA replication
Nuclear chromosome segregation
Mitotic sister chromatid segregation
Sister chromatid segregation
Chromosome segregation
Mitotic nuclear division
Organelle fission
DNA replication
Nuclear division
0 20 40 60
Count
p value
6e-11
4e-11
2e-11
B
Cellular component
Collagen-containing extracellular matrix
Condensed nuclear chromosome kinetochore
Condensed chromosome
Condensed nuclear chromosome, centromeric region
Extracellular matrix
Condensed chromosome kinetochore
Chromosomal region
Condensed chromosome, centromeric region
Kinetochore
Chromosome, centromeric region
0 20 40 60
Count
p value
5e-10
4e-10
3e-10
2e-10
1e-10
C
Molecular function
DNA replication origin binding
DNA-dependent ATPase activity
E-box binding
3'-5' DNA helicase activity
Single-stranded DNA-dependent ATP-dependent DNA helicase activity
DNA helicase activity
Core promoter sequence-specific DNA binding
Catalytic activity, acting on DNA
Cyclin-dependent protein serine/Threonine kinase regulator activity
Extracellular matrix structural constituent
0 5 10 15 20 25
Count
p value
5e-05
4e-05
3e-05
2e-05
1e-05
D
Pathway
Pyrimidine metabolism
Mismatch repair
p53 signaling pathway
Cell cycle
DNA replication
0 10 20
Count
p value
0.0020
0.0015
0.0010
0.0005
E
Biological process
Protein−DNA complex assembly
Mitotic cell cycle phase transition
Cell cycle phase transition
Nuclear chromosome segregation
Chromosome segregation
Mitotic sister chromatid segregation
Organelle fission
Mitotic nuclear division
Sister chromatid segregation
Nuclear division
0 20 40 60
Count
p value
3e−10
2e−10
1e−10
F
Cellular component
Condensed chromosome
Condensed chromosome, centromeric region
Spindle
Cytosolic large ribosomal subunit
Kinetochore
Extracellular matrix
Collagen−containing extracellular matrix
Cytosolic ribosome
Chromosomal region
Chromosome, centromeric region
0 20 40 60
Count
p value
8e−10
6e−10
4e−10
2e−10
G
Molecular function
rRNA binding
DNA replication origin binding
Growth factor binding
mRNA binding
Nucleosomal DNA binding
3'−5' DNA helicase activity
Helicase activity
Catalytic activity, acting on DNA
Extracellular matrix structural constituent
Structural constituent of ribosome
0 10 20 30
Count
p value
2e−05
1e−05
H
Pathway
p53 signaling pathway
Pyrimidine metabolism
Cell cycle
Ribosome
DNA replication
0 10 20 30
Count
p value
0.005
0.004
0.003
0.002
0.001

Fig. S3

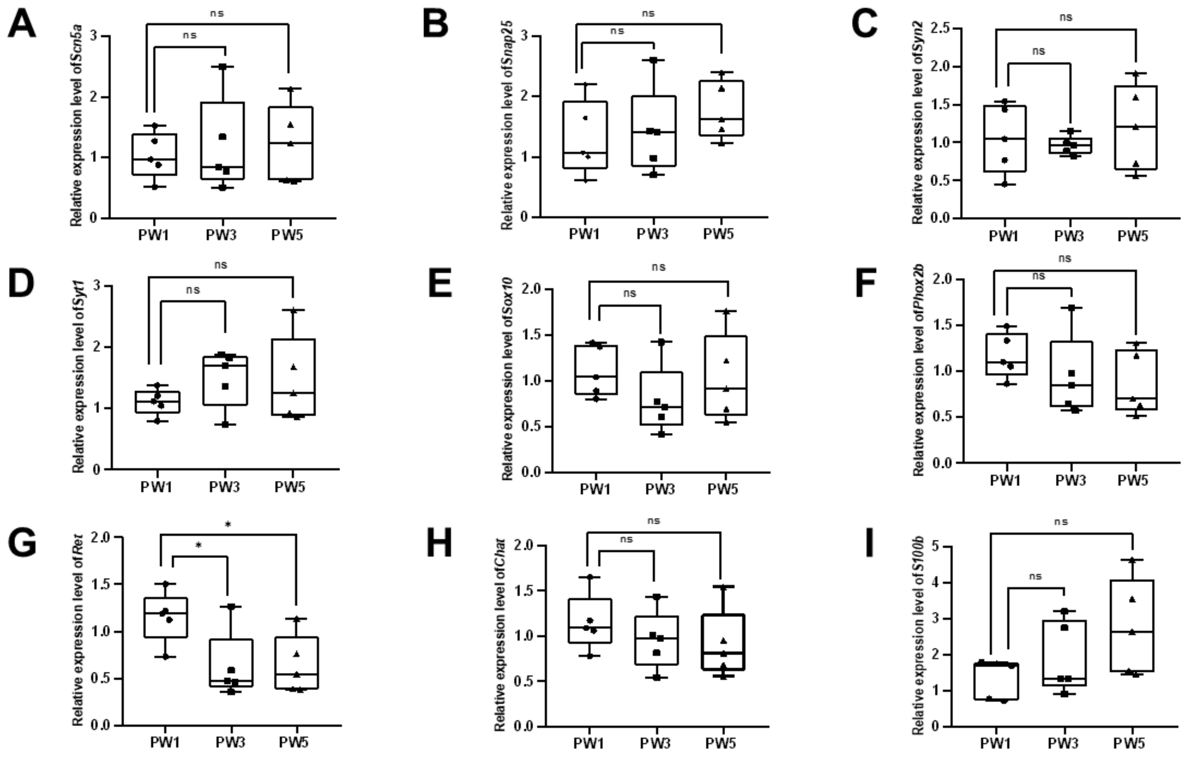
A
Relative expression level of Scn5a
ns
ns
PW1
PW3
PW5
B
Relative expression level of Snap25
C
Relative expression level of Syn2
D
Relative expression level of Syt1
E
Relative expression level of Sox10
F
Relative expression level of Phox2b
G
Relative expression level of Ret
*
*
H
Relative expression level of Chat
I
Relative expression level of S100b

Fig.S4

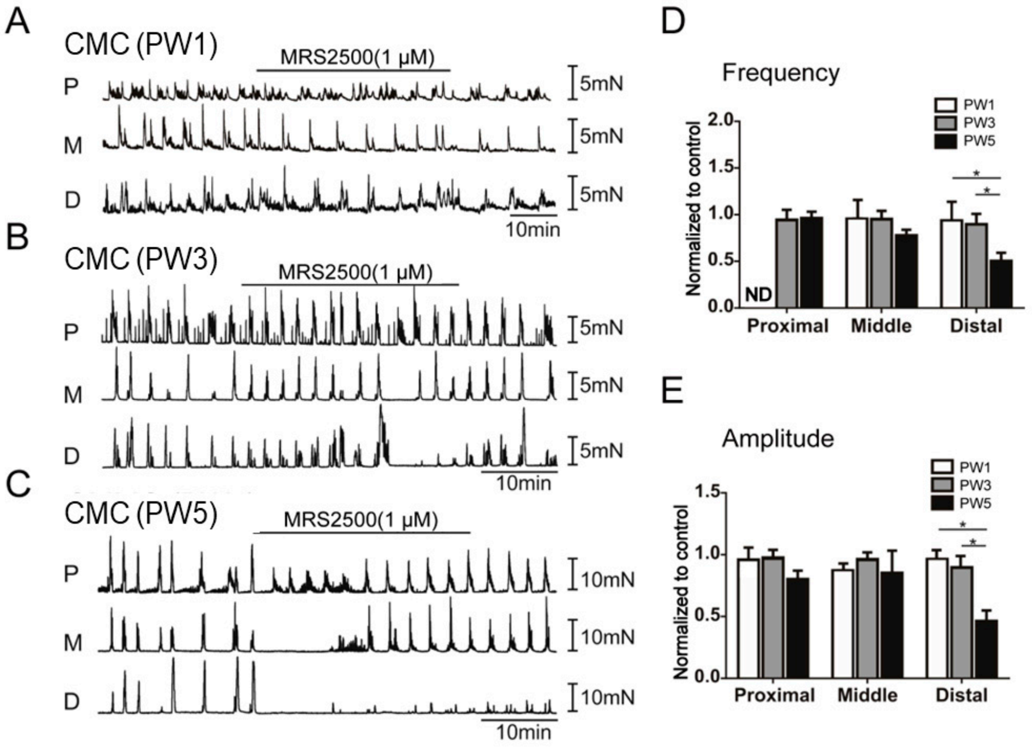
A
CMC (PW1)
MRS2500(1 μM)
P
M
D
5mN
5mN
5mN
10min
B
CMC (PW3)
MRS2500(1 μM)
P
M
D
5mN
5mN
5mN
10min
C
CMC (PW5)
MRS2500(1 μM)
P
M
D
10mN
10mN
10mN
10min
D
Frequency
PW1
PW3
PW5
Normalized to control
2.0
1.5
1.0
0.5
0.0
ND
Proximal
Middle
Distal
E
Amplitude
PW1
PW3
PW5
Normalized to control
1.5
1.0
0.5
0.0
Proximal
Middle
Distal
